# New Medical Journals

**Published:** 1853-02

**Authors:** 


*New Medical Journals.*

"The Southern Medical and Surgical Journal," edited by Drs. John W. King, W. P. Jones, R. O. Curry, and B. Wood, is published bi-monthly at Nashville, Tennessee. The editors give assurance that it shall live one year at least. Unless it should become weak and sickly, of which we see no indications in the first issue, we hope it may live long. If in a multiplicity of Doctors there is safety, we think the publishers need not fear. Price $2 per annum.

"The Memphis Medical Recorder" has been received. It is published bi-monthly by the Memphis Medical College, and edited by Professors Merrill and Quintard. It is filled to the utmost capacity of its 16 pages, with interesting and useful matter. Price $1 per annum.

"The Æsculapian," a monthly medical journal for the people, edited by C. D. Griswold, M.D., New York, has been received. Dr. G. has commenced well. The matter of the "Æsculapian," is such as will interest and profit, and we think it may do much good. As in everything else, so, in medicine, knowledge has been sought by some royal road—the end without labor. Quackery has accommodated itself to this condition of the public mind. To expose the fallacy of this idea, we want unfolded the science on which, as a superstructure, is raised the art of medicine; the people must see that prescribing for the sick is not altogether empirical, but that a knowledge of the laws of nature suggest to the educated physician the means for overcoming disease. We would be glad to see it in every family. Price $1 per annum.

